# The Impact of the COVID-19 Pandemic on Mental Health: Evidence from Cyprus

**DOI:** 10.3390/ijerph18083868

**Published:** 2021-04-07

**Authors:** Marilena Mousoulidou, Michailina Siakalli, Andri Christodoulou, Marios Argyrides

**Affiliations:** Department of Psychology, Neapolis University Pafos, 8042 Paphos, Cyprus; m.siakalli@nup.ac.cy (M.S.); andri.christodoulou@nup.ac.cy (A.C.); m.argyrides.1@nup.ac.cy (M.A.)

**Keywords:** COVID-19, first wave, pandemic, mental health, psychological impact, Cyprus

## Abstract

The aim of the current study was to examine mental health effects of the first wave of COVID-19 (Corona Virus Disease-19) in Cyprus. Accordingly, 388 individuals aged 18–65+ responded to the Components of Mental Health Questionnaire that was distributed via social media for two weeks assessing how emotional distress, support and interest in self and others, lifestyle changes, engagement in protective measures, and avoidant behaviors were related to participants’ gender, age, and place of residency. Additionally, we measured the level of concern of individuals during and after the first wave outbreak of the pandemic. The results suggest that (a) females experience higher levels of anxiety, stress, fear, worry, and despair than males and are more likely to undertake protective measures, (b) older individuals and those who live in urban areas perceive greater social support and interest in the emotional experience of significant others, (c) emotional distress and support and interest in self and others are associated with all other variables, indicating the importance of these constructs to the experience of a pandemic, and (d) there was a decrease in participants’ concern after the end of the first wave of the pandemic. Mental health professionals could find this information useful when developing and implementing prevention programs that aim to offer psychological support during this stressful period.

## 1. Introduction

The way people react and respond when facing a pandemic is a topic that has been of interest to the research community for decades. Whether gaining a greater understanding of human resilience or preparing for dealing with future outbreaks, researchers have often been intrigued by the impact a pandemic has. Past studies around previous pandemics, including SARS (Severe Acute Respiratory Syndrome), MERS (Middle East Respiratory Syndrome), Ebola, and swine flu have illustrated the various psychological and behavioral impacts. Research conducted on the psychological effects of these pandemics has indicated that stress, anxiety, fear, despair, and even depression and PTSD (Post-traumatic stress disorder) symptoms are common throughout the pandemic [1,2,3,4]. When a pandemic involves a highly communicable virus, social isolation and quarantine are commonly used measures to delay the spread of a virus. Isolation may be protective for one’s physical health during a pandemic outbreak; nevertheless, it can have a countereffect on one’s psychological well-being. Studies showed that strict and prolonged isolation measures can cause high levels of stress, both to the people who are asked to stay isolated in their house and those who need to be isolated in a medical setting [2,5]. For instance, Kim et al. [2] found that over 40% of patients isolated or quarantined during the MERS pandemic required psychiatric interventions. Similarly, in a review of quarantine’s psychological impact on previous pandemics like SARS and Ebola, Brooks et al. [6] found that the psychological costs of strict isolation measures to the population are high. Feelings of anger, confusion, psychological distress, depression, and anxiety were common feelings experienced by these studies’ participants (see [6] for a review).

Behavioral changes are also common during periods of a pandemic [6,7,8,9,10]. Fear of contamination may lead people to undertake avoidance behaviors, like avoidance of crowds and public transportation [7,8,9,11,12]. A concern of infection may influence personal habits concerning disinfection, such as frequent handwashing and the use of antibacterial spray [7,8,9,11,12]. These behavioral changes can be long-term for some people. For instance, in the study by Cava et al. [11] during the SARS pandemic, it was found that avoiding crowds and vigilant handwashing was present for many months for some, while for others these behaviors were adopted as permanent changes in their lives. 

In December 2019, an ongoing outbreak of a novel coronavirus known as COVID-19 occurred in China. On 11 March 2020, following 18,000 cases in 114 countries, the World Health Organization (WHO, Geneva, Switzerland) declared COVID-19 as a pandemic [13]. Currently, there are over 100 million people affected by the disease, and with new cases appearing every day, it is still unclear when the pandemic will come to an end. There is no doubt that the COVID-19 pandemic affected most people’s lives [12,14,15]. Because of COVID-19, most individuals need to change the way they live, work, study, and socialize. In combination with the strict isolation measures, these sudden changes influence an individual’s emotional and psychosocial well-being [12,16,17]. 

We are currently dealing with what appears to be the second wave of the pandemic, with a lot of countries further enforcing social distancing, travel bans, lockdowns and isolation, and quarantine measures. Since COVID-19 is a new coronavirus, there is ongoing research with regards to its psychological effects on people’s mental health. Importantly, there is a consensus that the pandemic’s psychological impact will have a profound effect on the mental health of the population [12,18]. 

Published research that examined the influence of COVID-19 on the community’s mental health clearly pinpoints its negative psychological impact [12,15]. For instance, the research conducted by Rodriguez-Rey et al. [15] during the early stages of the COVID-19 pandemic (March, 2020) on the population of Spain, indicated that 36.6% of the 3055 participants experienced psychological distress. Similarly, research conducted in Australia by Newby et al. [12] highlighted the impact of the pandemic on the mental health of individuals. These researchers found that the mental health of 78% of their sample deteriorated since the outbreak of COVID-19. 

Social support is essential during stressful situations. On the one hand, it positively affects one’s mental health, and on the other, it acts as a protective factor reducing the negative psychological impact [19,20,21,22]. Existing literature suggests that in periods of a pandemic, family and friends’ perceived support increases [8,23]. For instance, Lau et al. [8] asked participants to report the support they received from family and friends before the SARS pandemic outbreak, compared to the support they received two months following the outbreak. They found that 28.4% of participants reported increased support from friends (3.8% reported decreased support), and 39.1% of participants reported increased support from family members (1.7% reported decreased support). In addition, they found that for a large proportion of their participants, the expression of emotions towards others and interest in the emotions of others, increased in the period after the outbreak of the pandemic. According to Lau et al., the increase in social support acts as a significant “cushion” protecting participants from negative mental health impact [8] (p. 114). Similar results were obtained by Zhang and Ma [23], who examined perceived support from family and friends during the COVID-19 pandemic in China. These researchers found that 64.6% of participants reported increased support by friends and 63.9% reported increased support by family members. In addition, they also found that the expression and interest in the emotions of others increased during the outbreak of the COVID-19 pandemic. According to Zhang and Ma [23], the pandemic’s stressful impact may have been reduced by these factors.

The literature suggests that females are more susceptible to experience negative mental health impact, such as higher levels of distress, anxiety, and depression, during stressful situations [24,25]. More specifically, in a cross-sectional study of the UK population, Smith et al. [24] found that females had higher levels of poor mental health during March 2020. Similarly, in their research with the Indian population, Varshney et al. [25] found that females experience the pandemic’s greater psychological impact. This finding has been replicated in many countries, such as China [26,27], Turkey [28], Spain [15], Austria [29], and Australia [12]. The findings from the ongoing research of the non-profit international organization CARE International, which collected data from many countries concerning the impact of COVID-19 in challenging environments, further support this claim concerning females. CARE International has published a number of Rapid Gender Analysis Data that aimed to provide initial analysis on the Gender Impacts. A recently published report of CARE International [30] of almost 10,000 people in 38 countries shows the striking differences between men and women regarding mental health. According to CARE International’s findings, while both men and women report experiencing worry, anxiety, and overall emotional fatigue due to the pandemic, women (27% as compared to 10% of men) report suffering from anxiety, inability to sleep, loss of appetite and trouble completing everyday tasks. 

Additionally, the area of residency appears to influence the experience of the pandemic. Existing literature suggests that the risk of mental health problems varies depending on whether people live in rural or urban areas [28,31,32]. However, there are contradicting results in the literature, and therefore, it is still unclear whether residents in rural or urban areas are more negatively affected by the pandemic. For instance, Liu et al. [31] found that urban areas residents report higher mental health-related problems due to COVID-19. On the contrary, Schweda et al. [32] found that people in rural areas experience higher generalized anxiety due to COVID-19. Therefore, it is unclear which area of residency has a more significant impact on the population’s experience of a pandemic.

Age is another factor of interest in the literature that examined the psychological impact of COVID-19 on people’s mental health. From the beginning of the COVID-19 pandemic, it has been documented that the elderly face higher risks for severe illness or even death because of the pandemic [33,34]. Consequently, reports in the literature identify the need for implementing prevention strategies to protect the psychological well-being of the elderly [14,35]. Though the psychological impact of the pandemic would be expected to be more profound in this age group [36], studies that examined the elderly, as well as differences between age groups, suggest a different pattern of results [32,36,37]. Specifically, van Tilburg et al. [37] found that the mental health levels of the elderly in the period of COVID-19 pandemic remained unchanged, though loneliness increased. Similarly, Schweda et al. [32] found that anxiety decreases with age, although COVID-19 related fear is higher in older individuals. Vahia et al. [36] found that older adults had lower levels of trauma–or stress-related disorder, anxiety disorder, and depression disorder compared to younger age groups. Literature suggests that children, adolescents, and young adults are most affected by the COVID-19 pandemic [16,38,39]. Considering the evolving nature of the pandemic and its impact, research in this area is still explored.

Cyprus is a small island-country in the Mediterranean which is typically described as a collectivistic culture characterized by the warmth and hospitality of its citizens [40], the strong emotional ties between family members, the expression of feelings between friends and family, and the close social interaction between the citizens [41,42]. Therefore, a pandemic that enforces social distancing may greatly affect a culture such as Cyprus. During the first wave of the pandemic, several restrictions took place in Cyprus. Beginning from March 10th, there were strict restrictions in travelling abroad, private and public schools closed down, social distancing measures like the prohibition of mass gatherings, and cancellation of mass events were in effect [43]. On the 13 March 2020 these measures were extended [44] and from the 15 March 2020, entry to the country for any citizen was prohibited [45]. On 24 March 2020, further restrictions were implemented and Cyprus entered a complete lockdown where citizens were prohibited from “unnecessary” movements [46]. Cyprus started decreasing its strict measures from May 2020 onwards and by the time the current study was implemented, which was in July 2020, new cases of COVID-19 had declined dramatically and the restrictions and social distancing rules were decreased even further [47,48]. To our knowledge, there are no published findings on the mental health of the general community of Cypriots during the time of the first wave of the pandemic. 

The current study’s main aim was to examine the psychological impact of the COVID-19 pandemic on Cypriot citizens’ mental health. As this study is the first of its kind, our analyses were exploratory. For this study, the following variables related to mental health were of interest: (a) experience of anxiety, fear, stress, worry, or despair; (b) perceived support from family and friends, expression of emotions towards others, and interest in the emotions of others; (c) the time spent for relaxation, rest and other activities of interest; (d) avoidance behaviors, such as avoiding large gatherings of people; (e) engagement in protective measures, such as the use of antibacterial sanitizer and disinfecting surfaces. Based on the existing literature and the purpose of the study, five hypotheses were formulated:

1. There will be gender differences on the variables of interest. Based on previous literature, we expected that women will experience higher levels of negative mental health impact compared to men.
2. There will be age differences on the variables of interest. In line with existing research, we hypothesized that the negative impact will be higher for young adults.
3. There will be significant differences on the variables of interest based on the area of residency. Published findings do not unambiguously suggest which residence area may be more prone to negative psychological impact, therefore this hypothesis is still open to investigation.
4. There will be associations between all variables of interest.
5. There will be significant differences in the level of concern of individuals during the pandemic outbreak, as compared to their level of concern following the pandemic outbreak.

## 2. Materials and Methods

### 2.1. Sample

The sample included 388 participants, with an uneven distribution between genders. Specifically, the sample consisted of 111 males (28.6%) and 277 females (71.4%). Age of the sample ranged between 18 and 65+. Namely, 45 participants (11.6%) were between 18 and 25 years old, 95 participants (24.5%) were between the ages 26 and 32 years old, 117 participants (30.1%) were 33–40 years old, 78 (20.1%) were between 41 and 48 years old, 27 (7.0%) were 49–56 years old, 16 (4.1%) were between 57 and 65, and 10 participants (2.6%) were aged 65+. Educational level included 20 participants (5.2%) with a high school certificate, 20 participants (5.2%) with a diploma, 129 participants (33.2%) with a Bachelor’s degree, 195 participants (50.3%) with a Master’s degree, and 24 participants (6.2%) with a PhD. With regards to geographic location, 300 participants (77.3%) were from urban and 88 participants (22.7%) were from rural areas.

### 2.2. Measures

Demographic information: A demographic questionnaire was administered, which asked participants to provide their age, sex, education level, occupational status, and whether they belonged to the vulnerable group for COVID-19 (as defined by the Cyprus Government guidelines and was provided as a footnote). 

Level of Concern due to COVID-19: A five-question questionnaire was delivered to examine the level of concern of participants. These questions referred to two periods: Period A (during the outbreak of the pandemic), and Period B (the period the study was undertaken; July, 2020). These questions were rated on a 5-point Likert-type scale ranging from not at all (receiving a score of 1) to too much (receiving a score of 5). The internal consistency value (Cronbach’s alpha) for the current sample is 0.86.

Components of Mental Health Questionnaire: To measure the impact of COVID-19 on the mental health of Cypriot citizens, the Components of Mental Health Questionnaire (CMHQ) [49] was used. This measure is not used for a clinical diagnosis; therefore, it does not have a cut-off score for placing an individual in any diagnostic criteria. Rather, it identifies constructs related to mental health with higher scores indicating more symptomatology of the construct assessed. The CMHQ has five subscales. The Engagement in Protective Measures subscale includes 8 items measuring the degree of engagement in protective measures (e.g., washing one’s hands, disinfecting surfaces, use of hand sanitizer). The Lifestyle Changes subscale includes 5 items that measure changes to one’s lifestyle (e.g., time spent resting, relaxing, doing activities of interest). The Emotional Distress subscale is comprised of 6 items that measure negative emotional experience (e.g., anxiety, stress, fear, worry, despair). The Avoidant Behaviors subscale consists of 7 items that measure the degree of avoidance (e.g., public transportation, large gatherings, public places). The Support and Interest in Self and Others subscale includes 7 items that measure the expression and interest to self and significant others (e.g., expressing feelings, offering support). Items for the subscales Engagement in Protective Measures and Avoidant Behaviors are rated on a 5-point Likert-type scale ranging from very little (receiving a score of 1) to very much (receiving a score of 5) and participants are asked to report the behaviors they are undertaking during the current period. The items for the subscales Lifestyle Changes, Emotional Distress and Support, and Interest in Self and Others are rated on a 5-point Likert-type scale ranging from greatly increased (receiving a score of 1) to greatly decreased (receiving a score of 5) and participants are asked to report the level of concern they experienced before and during the outbreak of the pandemic. For the current sample, the Cronbach’s alphas ranged between 0.74 and 0.85. The internal consistency coefficient alpha for the Engagement in Protective Measures subscale was 0.85, for the Lifestyle Changes subscale was 0.77, for the Emotional Distress subscale was 0.74, for the Avoidant Behaviors subscale was 0.81, and for the Support and Interest in Self and Others subscale was 0.79.

### 2.3. Procedure

The study was approved by the Neapolis University Pafos institutional ethics and research board. The aforementioned measures were combined into one questionnaire which was created via Form Assembly, an online form builder. Data were collected via convenience and snowball sampling methods between the 15 and 31 July 2020. In an effort to have a representative sample and approach all districts of Cyprus and its citizens, the researchers in collaboration with The Cyprus Youth Clubs Organization, created a banner advertising the study and the link to completing the questionnaire. The banner along with the link, was distributed to all The Cyprus Youth Clubs Organization’s and the researchers’ contacts. Participants were also requested to forward the link to their own contacts. The social media platforms Facebook, Twitter, and Instagram, as well as the social chatting applications Viber, Messenger and WhatsApp were used for distribution. The banner and link were additionally promoted in the local online newspapers PafosPress and Paideia-News, as well as the Neapolis University Pafos and The Cyprus Youth Clubs Organization websites. All participants were informed of the purpose of the study, assured of their anonymity in participating, and consent was received when they submitted their final answers. All data were entered into the Statistical Package for Social Sciences, Version 25.0 (SPSS 25, IBM Corporation, Armonk, NY, USA) and all the necessary analyses were conducted. 

## 3. Results

Hypothesis 1 was partially supported. A Mann Whitney test was conducted to explore possible gender differences on all variables of interest. Statistically significant gender differences were obtained on the subscales Engagement in Protective Measures (*U* = 10239, *z* = −5.151, *p* = 0.000, effect size = 0.26) and Emotional Distress (*U* = 11881.5, *z* = −3.514 *p* = 0.0004, effect size = 0.178), where women had higher scores than men in both cases. For the subscale Engagement in Protective Measures, the reported mean for women was 213.04 while for men it was 148.24, and for the subscale Emotional Distress the respective values were 207.11 and 163.04. There were no statistically significant differences for all other variables of interest between men and women (*p* > 0.05).

Hypothesis 2 was also partially supported. The age categories were adjusted accordingly in order to reflect the three age categories i.e., early adulthood (18–40 years old), middle (41–64 years old), and late adulthood (65+ years old). The non-parametric Kruskal Wallis test was used to examine possible age differences on the five variables under consideration. The non-parametric test selection was based on the unequal sample size of the age groups and in one case of the low sample, the late adulthood. Statistically significant differences were obtained for the subscales Engagement in Protective Measures (*χ^2^* = 6.47, df = 2, *p* = 0.039), Avoidant Behaviors (*χ^2^* = 18.03, df = 2, *p* = 0.0001), and Support and Interest in Self and Others (*χ^2^* = 7.23, df = 2, *p* = 0.027). A post-hoc Dunn’s test was applied for all significant subscales. For the subscale Engagement in Protective Measures the Dunn’s test indicated that there are statistically significant differences between the early adulthood and late adulthood groups with late adulthood having higher mean ranks (M of ranks = 281.55) than the early adulthood (M of ranks = 190.17). For the subscale Avoidant Behaviors, differences were obtained between middle and late adulthood and early and late adulthood, where in both pairwise comparisons the age category late adulthood had higher scores (M of ranks = 331.1) than the other two (M of ranks = 204.83 for middle adulthood and M of ranks = 184.32 for the early adulthood). For the subscale Support and Interest in Self and Others the post hoc test indicated that statistically significant differences were observed between late adulthood and the other two. Namely, the age category late adulthood offered more support (M of ranks = 286.75) during the pandemic than middle (M of ranks = 195.6) adulthood and early adulthood (M of ranks = 190.4). No differences were obtained for the aforementioned subscales between the age categories early and middle adulthood.

Hypothesis 3 was also partially supported. A Mann–Whitney test showed statistically significant differences in the subscale Support and Interest in Self and Others and those living in urban and rural areas. For the subscale Support and Interest in Self and Others statistically significant differences (*U* = 11002.5, *z* = −2.93, *p* = 0.017, effect size = 0.007) were obtained with participants living in urban areas (Median = 60547.50, *n* = 300) having higher scores than the participants living in rural areas (Median = 14918.50, *n* = 88). For all other subscales, statistically significant differences based on the geographical division were not obtained *p* > 0.05.

Hypothesis 4 was also partially supported. Spearman’s correlation was used to explore possible associations between the five subscales (see Table 1). The results showed that the subscale Emotional Distress was positively related to all subscales except the subscale Lifestyle Changes, where the correlation was negative. That is, the higher the experience of fear, anxiety, worry or despair by the participants, the higher their engagement in protective measures, the more they avoided large gatherings, and the greater the expression of support and interest; the more time participants spent on relaxation and rest, the less distressed they felt. Furthermore, the subscale Support and Interest in Self and Others was positively associated to all other subscales. That is, the more perceived support, expression, and interest in the well-being of friends and family the participants had, the greater the engagement in protective measures, the more lifestyle changes were made, and the higher the avoidant behaviors were observed. Additionally, the results showed that there was a positive association between the subscales Engagement in Protective Measures and Avoidant Behaviors. That is, the more respondents engaged in protective measures, such as the use of antibacterial sanitizer, the more they engaged in avoidant behaviors, such as avoiding public transportations and travel. Lastly, the subscale Lifestyle Changes showed no relation with Engagement in Protective Measures and Avoidant Behaviors.

Hypothesis 5 was supported. A Wilcoxon signed-rank test revealed statistically significant differences on the level of concern for a personal infection of the participants between Period A and Period B (*z* = −12.68, *p* = 0.000) with medium effect size (r = 0.45). A reduction on the level of concern was observed after the outbreak with a median score during the outbreak (Median = 3) and after the outbreak (Median = 2). The same test was applied related to the level of concern of infection to the inner circle of the participants. The results obtained showed again that there is a reduction between these two periods of interest (*z* = −13.39, *p* = 0.000) with medium effect size (r = 0.48). The median score on the variable of interest decreased from during the outbreak (Median = 3) to after the outbreak (Median = 2). This can be justified by the fact that during the period that the survey was conducted, the level of COVID-19 cases had dramatically declined.

Multiple regression was used to explore the relative relationship between the overall concern of the participants related with the pandemic at the time that the survey was conducted (Period B) and the five subscales of CMHQ (Emotional Distress, Support and Interest in Self and Others, Lifestyle Changes, Engagement in Protective Measures, Avoidant Behaviors). Regression analysis could be applied as the residuals of errors were approximately normally distributed. The overall model was significant *R*^2^ = 0.31, *R*^2^*_adjusted_* = 0.30, *F* (5382) = 34.58, *p* < 0.05 (see Table 2). Overall, the Emotional Distress, Avoidant Behaviors and Engagement in Protective Measures subscales were related to the overall concern of the participants to the pandemic. Emotional Distress was the subscale that most affected the overall concern.

## 4. Discussion

The present study aimed to examine the psychological impact of the COVID-19 pandemic on the mental health of the population of Cyprus. To assess the impact of the COVID-19 pandemic to one’s mental health, we used the CMHQ [49] questionnaire that includes five subscales that measure: Emotional Distress, Support and Interest in Self and Others, Lifestyle Changes, Engagement in Protective Measures, and Avoidant Behaviors. In our analyses we examined how these factors were influenced by gender, age, and place of residency. Moreover, we examined the participants’ level of concern of infection due to the COVID-19 outbreak, by asking participants to respond to a five-question questionnaire that related to their experience during and after the period of the initial outbreak of the pandemic. Overall, our findings suggest that age, gender and place of residency influence the experience of the pandemic. The results of the current study could be used by researchers interested in possible factors that influence individuals’ mental health during a pandemic, as well as by mental health professionals when developing programs related to mental health care.

We first examined possible gender differences in the five subscales of the CMHQ. Our results indicated that women reported higher scores in the scales of Emotional Distress and Engagement in Protective Measures. Thus, our results replicated the well-documented findings in the literature which suggest that the experience of a pandemic is different for men and women [12,15,24,25,26,27,28,29]. The current study found that Cypriot women, compared to Cypriot men, reported higher levels of anxiety, stress, fear, worry, or despair. Therefore, women clearly experienced greater psychological impact due to the pandemic. Additionally, the current results showed that females displayed greater personal disinfection habits. That is, women were more likely than men to take preventive measures and engage in hygienic behaviors, such as using hand sanitizer and disinfecting surfaces. A possible explanation of this finding is that frequent disinfection may be a way in which women dealt with the threat of the spread of the virus, as well as the reported emotional distress they experienced regarding the outbreak. It should be noted though that the current results should be interpreted with caution considering that 71.4% of the participants were females as compared to the 28.6% of males. 

The literature suggests that age may be an influential factor in how one experiences life situations and in particular when facing a stressful situation like a pandemic. Our results showed significant differences between the three age groups (early adulthood, middle adulthood, and late adulthood) and the subscales Support and Interest in Self and Others, Engagement in Protective Measures, and Avoidant Behaviors. In particular, our findings suggest that people aged 65 and above showed statistically higher interest in how significant others experienced the pandemic as compared to the middle and the early adulthood participants. Despite the limited number of participants in this group, this finding appears to be showing the importance of social support in the lives of individuals in late adulthood [19,20,21,22]. Moreover, our results suggest that older individuals (late adulthood) engage in protective measures against the virus’s spread at a higher frequency than younger individuals, particularly those within the early adulthood group. Additionally, older individuals were also found to engage in avoidant behaviors at a higher frequency than both people in middle and early adulthood. A possible explanation of these findings is that older individuals tend to face greater health challenges placing them within the vulnerable group against the pandemic [33,34], leading to greater engagement in protective and avoidant behaviors. 

Based on the literature, children, adolescents, and young adults expressed greater psychological distress than other age groups [16,38,39]. As our research focused on young and older adults, we hypothesized that our findings would be in accordance with the current literature. Importantly, however, our results did not show significant differences between the three age groups and the Emotional Distress subscale. Therefore, this finding was not replicated. One possible explanation is the fact that our study, did not include children and adolescents, the groups that appear to be more severely negatively affected by a pandemic. Another possible explanation is the small sample size of late adulthood participants and as such the results might not be representative of the group as a whole. Since no significant differences were noted within the different age groups, it suggests that participants’ experience of the pandemic may be similar and therefore mental health care should be directed to all regardless of age. 

Another interesting result comes from examining how the area of residency affects the five subscales related to mental health. The current results demonstrated significant differences in whether individuals resided in urban or rural areas and the subscale Support and Interest in Self and Others. That is, residents in urban areas reported greater perceived emotional support from friends and family, expressed their emotions towards significant others in greater frequency, and showed more interest in the emotions of significant others during this period. This is an important finding since the literature agrees that social support positively affects one’s mental health and can act as a protective factor reducing negative psychological impacts [19,20,21,22]. Despite the challenges of COVID-19, it appears that the pandemic offered the opportunity for individuals from urban areas to reconnect with self and others and to express themselves more openly regarding their experiences. This suggests that during periods of crisis people may turn to others for support. As Zhang and Ma [23] stated, during a pandemic, the pace of a whole society slows down, creating greater opportunities and time amongst community members to support and care for one another. 

It is worth noting, that our results did not demonstrate significant differences in the other subscales with regards to place of residency. This could suggest that people who live in urban areas and those who live in rural areas experience similar mental health impacts due to the pandemic. This finding can be explained by the fact that Cyprus is a small island country and thus rural and urban areas have a close proximity to each other and might be experiencing similar effects. Therefore, place of residency appears to be inconsequential to the need for psychological support in periods of a pandemic.

The examination of possible associations between the five factors of the CMHQ showed that the subscale Emotional Distress was weakly, yet significantly associated with all factors of interest. Specifically, it was positively related to the subscale Engagement in Protective Measures, Avoidant Behaviors and Support and Interest in Self and Others, and negatively related to Lifestyle Changes. That is, the higher the experience of stress, fear, anxiety, worry or despair by the participants, the higher their engagement in protective measures, the more they avoided large gatherings, and the greater the expression of support and interest. Moreover, the more time participants spent on relaxation and rest, the less distress they felt. It appears that the way individuals cope with the threat of a pandemic may influence the way they behave. Furthermore, the subscale Support and Interest in Self and Others was also weakly, yet significantly associated to all variables of interest. That is, the more perceived support, expression, and interest in the well-being of friends and family the participants had, the greater the engagement in protective measures, the more changes they made to their lifestyle, and the more they displayed avoidant behaviors. 

Additionally, the results showed a moderate positive significant association between the subscales of Engagement in Protective Measures and Avoidant Behaviors. That is, the more the respondents engaged in protective measures, such as the use of antibacterial sanitizer, the more they engaged in avoidant behaviors, such as avoiding public transportations and travel. Engaging in protective and avoidant behaviors which are the proposed necessary measures to minimize the spread of COVID-19 [33], may be indicative of an individual’s awareness of the risks of the virus. This interpretation is supported by past research that found that perceptions of personal health risk can predict engagement in protective behaviors against the spreading of the virus [50].

Of particular concern and interest was people’s reported experiences during and following the outbreak in order to specify the challenges they faced. Participants were asked to report their level of concern during the outbreak (between March and April 2020 where the Cyprus Government forced several restrictions, including the lockdown) and their level of concern at the time of the study (July 2020). Participants’ reports provided an exploratory measure of the changes in the level of concern they experienced. Our findings show that participants’ level of concern for their personal safety and the safety of their significant others, declined. One potential explanation for this finding is the timing of the current study. It is possible that the severity of the pandemic was underestimated as it was at the end of the first wave, signified by the reduction of reported positive cases as well as the easing of government restrictions. These changes might have influenced participants to believe that the pandemic was over, thus lowering their level of concern.

Further investigations of the overall concern of the participants showed that the level of concern was impacted by the Emotional Distress subscale, followed by the Avoidant Behavior subscale, and lastly by the Engagement in Protective Measures subscale. This appears to be suggesting that anxiety, stress, fear, worry, or despair might have influenced the overall level of concern of individuals. It seems important therefore, for intervention programs to focus on ways of coping with emotional distress which will simultaneously help individuals reduce their level of concern when facing unexpected events such as a pandemic.

Researchers and mental health professionals could find the results of the current research useful during this challenging period of the COVID-19 pandemic. Our results suggest possible factors that influence individual’s mental health which could be considered when implementing counselling services. We believe that there is a need for implementing widely available counselling services, which are affordable and available to both those infected with COVID-19 and their families, and the general public. For these services to be accessible to the general population, it is recommended that they be digitally available, telephone- and- internet-based, enabling those in need to seek psychological help without necessarily leaving their space. We consider that these services will help outweigh the negative mental health impact of the pandemic. Future research should focus on examining the level of concern of participants, especially during this period that appears to be the second wave of the pandemic. Our results showed that the level of concern decreased when the population returned to “normality” and it is important to examine the reason behind it. Furthermore, additional research could focus on specific mental health constructs (e.g., depression, anxiety) using standardized clinical tools for a thorough examination of clinical symptomatology associated to COVID-19.

Our study is an important contribution to the literature as it investigates the psychological impact of the COVID-19 pandemic on Cypriot citizens’ mental health in Cyprus. Nevertheless, the results of the current study should be viewed within the context of the following limitations. Firstly, due to the fact that snowballing methods and social media were used to collect the data, any generalization statements reported should be interpreted with caution. The same caution should be used with the constructs mentioned in the study. Moreover, the relatively small sample size impedes upon the generalizability of the results and the age categories were not proportionately formed due to the unequal number of people within each category. Specifically, the sample size of the late adulthood was very small in comparison to the other categories, which resulted in an underrepresentation of this age category. Also, the period that the survey was undertaken is not representative of the actual level of concern as the restrictive measures were loosened and the majority of the people had returned to “normality”. In addition, the current research included questionnaires of self-reporting which poses concerns on the objectivity of the answers of the participants. Lastly, our participants were asked to remember information, which increases the possibility of recall bias from individuals. 

## 5. Conclusions

In the present research, we examined the impact on mental health of Cypriot citizens during the first wave of the pandemic. The results from 388 individuals show that women, as compared to men, experience higher levels of anxiety, stress, fear, worry or despair, and are more likely to undertake protective measures. Our results suggest that age and place of residency do not influence how individuals experience negative psychological effects. However, there are differences in the way they perceive social support and interest in the emotional experience of significant others. Importantly, the variables Emotional Distress and Support and Interest in Self and Others are weakly yet significantly associated with all other variables, indicating the importance of these variables to individuals’ experience of a pandemic. Lastly, our study suggests a decrease in our participants’ concern after the end of the first wave of the pandemic. Mental health professionals aiming at psychological support and interventions during this stressful period, could consider all of the above when implementing programs that aim to reduce the negative impact of the pandemic.

## Figures and Tables

**Table 1:** Correlation among CMHQ subscales.

Variables	1	2	3	4	5
1. Emotional Distress	------				
2. Support and Interest in Self & Others	0.282 **	------			
3. Lifestyle Changes	−0.191 **	0.190 **	------		
4. Engagement in Protective Measures	0.176 **	0.192 **	0.035	------	
5. Avoidant Behaviors	0.267 **	0.175 **	−0.070	0.466 **	------

Note. Total *n* = 388. ** *p* < 0.01.

**Table 2:** Results of multiple regression analysis predicting the level of concern.

Variables	B	S.E.	*β*	t
Constant	−0.583	0.381		−1.533
Emotional Distress *	0.065	0.013	0.241	5.134
Support and Interest in Self & Others	0.002	0.013	0.006	0.128
Lifestyle Changes	−0.003	0.012	−0.012	−0.268
Engagement in Protective Measures *	0.018	0.007	0.119	2.397
Avoidant Behaviors *	0.056	0.008	0.365	7.270

Note. * *p* < 0.05.

## Data Availability

No public data is available for this study.
